# Preprint pointers from a long COVID scoping review: considerations for source selection and searching

**DOI:** 10.29173/jchla29741

**Published:** 2024-08-01

**Authors:** Sarah C. McGill

**Affiliations:** Research Information Specialist, Research Information Services, Canadian Agency for Drugs and Technologies in Health (CADTH), Ottawa, ON

## Abstract

This paper describes the search approach for preprints for a post COVID-19 condition (i.e., long COVID) scoping review, including source selection, search strategy development, challenges, and insights throughout a project life cycle. With the growth of medical preprints since the COVID-19 pandemic, information professionals and researchers should be aware that preprints are possible sources of evidence and be prepared to manage them in evidence reviews for COVID-19 topics and beyond. Preprints are not peer-reviewed but can include important evidence about emerging topics. Because of the importance of preprints to the scoping review, a preprint search of Europe PubMed Central (PMC) was added. Europe PMC and similar aggregators combine multiple preprint servers and often have Boolean search, but sometimes limited search functionalities or few export options. Strategy translation encountered challenges such as varying and inconsistent terminology for post–COVID-19 condition, a complex search, and negotiating large numbers of preprints with resource constraints. Europe PMC identified additional preprints for inclusion due to additional preprint server coverage. It was helpful to limit the preprint search to the title and abstract fields, and to run an extra Internet search for publication of included study preprints. Challenges and potential solutions are summarized to support those conducting preprint searches for COVID-19 and other topics.

## Introduction

### Rationale for this paper

This paper describes the search approach for preprints for a post COVID-19 condition (i.e., long COVID) scoping review, including source selection, search strategy development, and insights for scoping or systematic review teams. With the growth of medical preprints since the COVID-19 pandemic, information professionals and researchers should be aware that preprints are possible sources of evidence and be prepared to manage them in evidence reviews for COVID-19.

### Preprints and the COVID-19 pandemic

The COVID-19 pandemic brought preprints to attention for rapidly sharing research developments, often months before final publication [1-3]. A preprint is an open-access version of a scientific manuscript before peer review that has been posted to a public repository or server. Many publications have highlighted the role of preprints in the COVID-19 pandemic. Early on, Shokraneh and Russell-Rose (2020) [4] described COVID-19’s terminology chaos and suggested evidence syntheses include preprint servers. Gianola et al. (2020) [5] examined relative proportions of COVID-19 publication types including that there were more preprints than published articles on COVID-19 at the time. Flanagin et al.’s (2020) editorial [6] discussed preprints’ benefits and challenges, and summarized journals’ author guidance. Notably, Fraser et al. (2021) [3] emphasized the unprecedented role of preprints in evidence dissemination, and that preprints were shared more and reviewed faster than previously. Thomas’ (2021) [7] preface to JEAHIL’s COVID-19 issue highlighted innovative evidence surveillance projects mapping publications and preprints. Recently, Blatch-Jones et al.’s (2023) scoping review [8] has explored the use and acceptability of preprints broadly across health and social sciences.

As preprints have not been peer-reviewed, information professionals and researchers may be apprehensive about including them in evidence syntheses. However, there are benefits to including preprints. The *Cochrane Handbook for Systematic Reviews of Interventions* describes preprints as a potential source for systematic reviews [9]. Including preprints reduces the risk of bias resulting from missing evidence. Preprints are also beneficial for early awareness of overlapping research. For example, our project team conducted an exploratory search for reviews, including preprints, to reduce research waste. Of particular interest, Clyne et al. (2021) [10] describe lessons learned from incorporating preprints in multiple COVID-19 rapid reviews. As we echo later in this paper, Clyne highlights that review teams should have a clear protocol for selecting sources, consider a sensitivity analysis of the impact of including preprints, label preprint included studies, and plan a checkpoint for finding and matching their subsequent peer-reviewed publications. Brody et al.’s (2023) paper [11] in the *Journal of the Medical Library Association* provides best practices for searching for COVID-19 and other public health emergencies including preprints.

Other publications have reviewed preprint search aggregators including Europe PMC [12] and the preVIEW COVID-19 preprint search [13-16]. Kirkham et al. (2020) examined medical and biomedical preprint platforms that were launched up to 2019 [17] and Wikipedia maintains a multidisciplinary list [18]. Online Supplement, Appendix 1 lists the main preprint aggregators for health.

Supplement, Appendix 1

The pandemic has inspired research monitoring the evidence quality of preprints, including changes post peer review, and found that most conclusions remain consistent after publication [8,19-24]. However, a small percentage of published preprints may have changes to outcomes, sample sizes, and results [19]. Of the preprints in a study by Bai et al. (2023) [19], those with a small sample size or high risk of bias were less likely to be published in a journal. Overall, about 25% of the preprints remained unpublished after 2 years.

### Context of the CADTH long COVID scoping review

Motivated by a national concern for COVID-19 patients with ongoing symptoms, the Canadian Agency for Drugs and Technologies in Health (CADTH) undertook a scoping review [25] in 2021 to quantify the available evidence on post COVID-19 condition, also known as post-acute sequelae of severe acute respiratory syndrome coronavirus 2 (SARSCOV-2) or long COVID. Approximately 10% to 20% of patients with COVID-19 had prolonged or recurring symptoms [26,27], and CADTH and healthcare decision-makers in Canada needed to better understand this new condition. As a result, CADTH initiated a 2021 newsletter article [28] then a scoping review [25]. The reports were published on the CADTH website and in the Canadian Journal of Health Technologies [29,30]. The scoping review has since been helpful as an inventory of post–COVID-19 condition studies and in guiding new reviews.

The scoping review covered publications on COVID-19 clinical classifications, diagnosis, prognosis, prevention, treatment, and management for people of all ages in any setting, as well as evidence gaps. The initial review was published in May 2022 [29]. Updates to the scoping review in December 2022 onwards were targeted to treatment and management to reduce duplication of efforts internationally [30].

To capture as much evidence as possible, the May 2022 scoping review included a wide range of publication types: any comparative and noncomparative primary studies of any design; evidence syntheses; clinical guidelines; economic evaluations; and ethical analyses, including protocols, trial registries, conference abstracts, and preprints [29]. This project would be CADTH’s first scoping review and was ambitious in including all evidence types. Our team expected the topic to evolve rapidly and knew preprints would be useful in illuminating the scope of evidence and work in progress.

The main literature search included six bibliographic databases: MEDLINE, Embase, APA PsycINFO, the Cochrane Central Register of Controlled Trials (CENTRAL), and Philosopher's Index for an ethics search, all via the Ovid platform; and CINAHL (the Cumulative Index to Nursing and Allied Health Literature) via EBSCO. The search strategy was comprised of controlled vocabulary such as the National Library of Medicine’s MeSH (Medical Subject Headings), and keywords. The main search concept was post COVID-19 condition, as well as its synonyms. Parts of the strategy were adapted from CADTH’s COVID-19 search string [31]. The grey literature search was conducted using the CADTH Grey Matters checklist for COVID-19 [32], and clinical trial registries were also included. Final search strategies are outlined in the scoping review report [29].

### Early COVID-19 evidence surveillance

At the time of the 2021-22 scoping review, preprints had a limited presence in bibliographic databases. In mid-2020, Shokraneh and Russell-Rose [4] had written that none of the main bibliographic databases included preprints, to the detriment of COVID-19 synthesis efforts. By late 2021, MEDLINE included just preprints affiliated with the National Institutes of Health (NIH) [33] and Embase included preprints from medRxiv and bioRxiv [34]. However, other preprint servers were relevant to post−COVID-19 condition, including SSRN and PsyArXiv. Because of the importance of preprints, we added a search of preprints in Europe PMC [35].

In 2021, the language around post–COVID-19 condition was inconsistent and evolving, and new publications, protocols, and preprints were being published daily. For a sense of the volume, the May 2022 review found that, on average, one new post COVID-19 study became available daily [29]. Authors used terms including long COVID, long haulers, post COVID-19 condition, post SARS–CoV–2 syndrome, and post-acute sequelae of COVID-19 or coronavirus. Many publications did not use a specific term at all, instead describing cohorts or long-term outcomes following COVID-19 infection. This required strategic searching to reduce results on other long-term conditions, and careful screening to determine if patients were followed past 8-12 weeks. The WHO’s clinical case definition of post COVID-19 condition as persistent or new symptoms for 12 or more weeks [27] was published in October 2021, which helped coalesce definitions as we developed our protocol and search approach.

## Description

### Europe PMC preprints

A source that aggregates multiple preprint servers is key for efficient searching. Examples of free preprint aggregators are Europe PMC [35], OSF Preprints [36], Google Scholar (though there is no preprint filter), and PreVIEW [37]. As mentioned, MEDLINE [33] and Embase [34] include limited preprints. Recently, the subscription database Web of Science has added multiple preprint servers [38]. Europe PMC is hosted by EMBL-EBI, the European Molecular Biology Laboratory’s European Bioinformatics Institute, in partnership with PubMed Central. Alongside journal articles, it has a vast number of preprints (more than 709,000 as of December 2023) [35]. Europe PMC includes the preprint servers medRxiv, Authorea, arXiv, bioRxiv, ChemRxiv, F1000, Preprints.org, PsyArXiv, Research Square, SSRN, and others [35]. Features include limiting results by publication language, saving searches, sending email alerts, and downloading results in RIS, XML, and CSV. There are field codes for targeted searching, such as limiting keywords to the title and abstract (TITLE_ABS) [39]. For those familiar with API (Advanced Programming Interface), Europe PMC has annotations (terms for diseases, drugs, gene/protein names, and more) identified by text-mining algorithms: these are useful as controlled vocabularies for identifying articles on a topic, for those with programming experience [40,41]. Rosonovski et al. (2023) [12] introduce the basics of Europe PMC including searching for preprints. Information specialists will want to refer to Europe PMC’s Help for advanced search syntax [39].

### Preprint source selection criteria and rationale for selecting Europe PMC

Europe PMC’s coverage of many multidisciplinary preprint servers, including SSRN and PsyArXiv, and its use of traditional journal database search features made it easy to fit into our workflow. Its search functionality was flexible for our complex search and the full set of results could be batch downloaded to EndNote. An overview of the criteria used to evaluate Europe PMC for the scoping review is provided in Online Supplement, Appendix 2.

Supplement, Appendix 2

Other potential preprint sources were eliminated due to lesser search functionality. For example, OSF Preprints does not offer a way to download an entire batch of results. Google Scholar includes preprints and can be searched informally using a string to limit the search to preprint sources, but it does not allow for complex Boolean searches with reproducible results. The exception was Web of Science, which CADTH does not have a subscription to, and preVIEW. PreVIEW was discovered after the initial search; it has a comparable number of COVID-19 preprints to Europe PMC.

## Outcomes of preprints search

### Narrative description of preprint search

Preprints were identified for the scoping review by searching Europe PMC, bibliographic databases, and grey literature. An information specialist developed and conducted the preprint search strategy, adapted from the peer-reviewed search strategy for Ovid MEDLINE. The main search concept was post COVID-19 condition and its synonyms.

Due to resource constraints, the preprint search was limited to systematic reviews. A typical approach for searching a platform with less functionality is to broaden the search; however, thousands of preprints mentioning long COVID made this impractical. The information specialist discussed further options with the team, such as screening more preprints (which would require more time or screeners), searching fewer servers, or limiting by study design. The team’s past experiences showed most long COVID primary studies were case studies or series. Limiting to preprints for systematic reviews seemed low risk because case studies and small case series were of lower interest, the scoping search would be updated every 3 months, and the study designs summarized within the scoping review could be reported transparently for readers. We do not know for sure how limiting the preprint search to systematic reviews affected our understanding of the evidence, aside from a smaller count of included preprints, because the actual number of eligible preprint primary studies is unknown.

To limit the preprint searches to systematic reviews, the CADTH systematic review filter [42] was adapted for Europe PMC. Searches were limited to the title and abstract fields. This brought the results down to a manageable number.

Translating the numerous phrases for post COVID-19 condition to Europe PMC syntax was time consuming. Strings using adjacency were changed to phrases in quotes. At the time, Europe PMC appeared to have a search character limit, so the search was broken into 4 separate searches, with duplicates removed later in EndNote. Refer to Online Supplement, Appendix 3 for the Europe PMC search strategy.

Supplement, Appendix 3

The initial search was completed on October 15, 2021. Search strategies were saved within Europe PMC. The database and preprint searches were updated regularly until December 20, 2021.

**Key takeaways on searching for preprints**

- Preprints are a potential source of evidence for evidence syntheses.
- Review protocols should have clear inclusion criteria and methods for handling preprints.
- Plan preprint searches early. Estimate result numbers and impact on timelines.
- Seek guidance from an information specialist or librarian for all searches.
- Evaluate if the main database search sources include preprints (Medline, Embase, Web of Science) and if a separate preprint search is needed.
- Preprint aggregators like Europe PMC allow complex searching across several servers.
- Clearly label preprints throughout the project for the team and readers of the report.
- Plan a final publication check for all included preprint studies both on the server and the Internet.

### Citation management and study tracking

Europe PMC records were edited in EndNote using Move Field, so that the server’s name (e.g., medRxiv) appeared in the journal name field, to better deduplicate with preprints from Ovid. All preprints were labelled as “Preprint” in the Label field (as versus in the title) so as not to interfere with duplicate detection. Labelling also helped identify preprints from less familiar servers. The team found duplicates between the preprints and published articles, and it took time to identify their relationships using bibliographic information or trial registration numbers. Because published preprints sometimes changed titles or authors, the information specialist created a tracking document (a simple list of preprint citations with the final journal citation underneath) to help notify the team. Every search update, new items on this list were emailed to the team. This was redundantly documented in EndNote by adding “Published” to the Label field and the journal citation to Notes. Published preprints were removed as duplicates in the screening software.

### Screening and charting (data extraction)

The same screening and charting forms were used for all retrieved citations including preprints, with the same double reviewer consensus methods. The screening form had separate variables for publication status and study design, so for example sources could be selected as both a systematic review and a preprint. For full details on screening and extraction, please refer to the published scoping review [29].

### Outcome: number of included preprints

The included studies, their characteristics, the PRISMA flow chart, and visualizations are published in the scoping review [29]. In total, there were 892 included studies, including 12 preprints of systematic reviews, plus 3 systematic review protocols published as preprints, for a total of 15 preprints [43]. 95 preprints were screened for the review, including 47 from the initial search (20 from Ovid MEDLINE and Embase, 26 from Europe PMC, and 1 from grey literature) and 48 from alerts across all sources.

Europe PMC found all 15 included preprints in the May 2022 scoping review. The MEDLINE and Embase searches found potentially relevant preprints, but these were published in subsequent search updates. Most included preprints came from medRxiv (10 of the 15, or 67%). The rest of the preprints came from Research Square (2), F1000 (2), and SSRN (1).

Revisiting these preprints two years later, all but three of the 15 included preprints have since been peer-reviewed and published (80% peer-reviewed and published; versus 20% not peer-reviewed, including one protocol, as of December 2023). Of the twelve peer-reviewed and published preprints, the preprint server linked to the final version for 75% (9 of 12), and the remaining 25% were found by Internet searching. This highlights that server linking is not perfect, and that double checking for published preprints by Internet searching is important.

## Discussion and recommendations for preprints in reviews

### Implications for preprints in the long COVID scoping review

The goal of the post COVID-19 condition scoping review was to understand the scope of a rapidly evolving topic. Several preprints were included in the 2022 scoping review, and most were published by 2023. The post COVID-19 condition scoping review has continued to have an impact as a starting point for new queries.

### Challenges of including preprints

It was complex to decide between searching fewer preprint sources (i.e., only preprints in MEDLINE and Embase) versus spending time translating the search strategy to Europe PMC and then narrowing the search because of so many preprint results. Europe PMC found unique preprints beyond the main search. However, time was required for strategy adaptations. Translation tools might be used in future to find the closest matching syntax in SR-Accelerator’s Polyglot [44] followed by a Find & Replace in a word processor.

Including preprints took more time overall and required an organized approach. Like any added source, they added source-specific steps and increased total results. Preprint sources often have less sophisticated search options, and the search must be translated to a different syntax and possibly modified. Especially if the search must be broadened, more results add screening and data extraction time. Because the final publication may not be automatically linked on the preprint server [10] or come into alerts, time is needed for a final check for publication. Clyne et al. (2021) [10] suggest scheduling a checkpoint to search for publications of all included preprints. Online Supplement, Appendix 4 provides a summary of preprint considerations and challenges discussed here, including protocol development and searching for and managing preprints.

Supplement, Appendix 4

### Limitations

Sources for preprints are evolving quickly, so features may have changed since the writing of this paper. Every month, a source may add new preprint servers or new search features. On a positive note, we found that the developers of Europe PMC and PreVIEW were open to improvement suggestions.

This paper focused on searching for a large COVID-19 topic and scoping review. Narrower topics may find searching larger preprint databases (like OSF Preprints) valuable and the number of results is manageable for manual download. Scoping reviews look at the amount of literature, and ours did not assess quality or risk of bias of the preprints.

We were also limited by budget and API programming knowledge. It would be worthwhile to investigate subscription databases with many preprints, like Web of Science; as well as to investigate options for accessing preprints by API, such as from OSF Preprints or OpenAlex [45].

### Directions for Future Research

The preprint environment is steadily changing, with more journals adding the option to publish preprints at the submission stage [46,47] and more repositories adding preprint servers to their searches. Future research is planned to evaluate preprint aggregators against selection criteria, including coverage and search functionality. We would like to better understand, for example, whether searching preprints in Embase can serve as a substitute for searching medRxiv and bioRxiv, and what is the best free preprint search option.

Future research could clarify options for including preprints in different types of reviews and beyond COVID-19 topics. Incorporating preprints is important for a scoping review to summarize the full landscape of publications. It would be beneficial to explore the experiences of other review teams. For example, was our decision to limit preprints to systematic reviews the best option or could we have searched fewer preprint servers? If specific medical disciplines publish more preprints than others (possibly bioinformatics or genetics [48]), advice on which disciplines benefit from searching preprints would be helpful. There may also be opportunities for improving preprint search features and standards for linking to the final publication.

## Conclusion

Preprints share emerging research developments months before the final publication, and their production exploded during the COVID-19 pandemic. Sources such as Europe PMC aggregate more preprint servers than most traditional bibliographic databases and found unique preprints for the post COVID-19 scoping review beyond the main database search. However, added time was required to adapt the strategy, screen additional results, and track published preprints.

In the long term, the work done for this scoping review will continue to inform future preprint searches at CADTH. We hope this paper will help others better prepare for searching and managing preprints in review.
