# Supplementary material for: Preprint pointers from a long COVID scoping review: considerations for source selection and searching
Source: J Can Health Libr Assoc. 2024 Aug 1;45(2):88–97. doi: 10.29173/jchla29741 (PMC11485164; doi:10.29173/jchla29741)
Supplement: Supplementary file 1 [file JCHLA-45-088-s001.pdf]

# Appendix 1- Main medical preprint search aggregators

| Name of preprint aggregator | Free or subscription | Number of preprint servers |
|---|---|---|
| **PreVIEW**<br><br>https://preview.zbmed.de/ | Free. | 9 preprint servers including medRxiv, bioRxiv, psyArXiv, SSRN. |
| **Europe PMC**<br><br>https://europepmc.org/ | Free. | Over 30 preprint servers including medRxiv, bioRxiv, psyArXiv, SSRN. |
| **Medline; PubMed**<br><br>https://pubmed.gov/ | PubMed – Free.<br><br>Subscription required for other platforms. | medRxiv, bioRxiv, arXiv, Research Square.<br><br>NIH-affiliated preprints only. |
| **Embase** | Subscription required. | medRxiv, bioRxiv only. |
| **Google Scholar**<br><br>https://scholar.google.com/ | Free. | Over 30 – crawls web. Including medRxiv, bioRxiv, psyArXiv, SSRN. |
| **OSF Preprints**<br><br>https://osf.io/preprints/ | Free. | Over 30 preprint servers including medRxiv, bioRxiv, psyArXiv, SSRN. |

| | | |
|---|---|---|
| **Web of Science** | Subscription required. | arXiv, bioRxiv, chemRxiv, medRxiv, Preprints.org.<br><br>May add more servers (Source). |

Preprint sources not included: Cochrane CENTRAL had under 100 preprints as of December 2023. Scopus is not listed because preprints are included only in its Secondary Documents (citation sources), not the main Scopus database. Collabovid.org is no longer updated. The COVID-19 Open Research Dataset (CORD-19) stopped updates in 2022. LitCOVID excludes preprints.
