# Supplementary material for: Preprint pointers from a long COVID scoping review: considerations for source selection and searching
Source: J Can Health Libr Assoc. 2024 Aug 1;45(2):88–97. doi: 10.29173/jchla29741 (PMC11485164; doi:10.29173/jchla29741)
Supplement: Supplementary file 2 [file JCHLA-45-088-s002.pdf]

Appendix 2 - Evaluation criteria

Evaluation criteria checklist for Europe PMC for the Long COVID scoping review. Shaded rows were must-have criteria for this review.

Evaluation criteria	Long COVID scoping review requirements	Europe PMC
Cost.	No additional costs.	✓ Free.
Preprint servers included.	Must have at least medRxiv and bioRxiv; preferably PsyArXiv and SSRN.	✓ Over 30 preprint servers.
Boolean search.	Must have for combining all long COVID synonyms with OR.<br>Combining concepts (AND) ended up being important for narrowing the search.	✓
Field search (e.g., limit terms to the title or abstract).	Must have if the full text is searched by default, because long COVID was often mentioned once in the full text or references.	✓ Fields codes for title, Abstract, Title_abs, date, and more.

Batch download.	Must have for large searches.	✓
Currency.	Must have regular updates.	✓ Updated daily.
Date limits.	Nice to have; alternatively, date cutoffs could be applied in the citation software.	✓ Limit by year.
Limit to only preprints, if the database includes non-preprints.	Nice to have; otherwise remove duplicates in the citation software.	✓
Save searches or alerts.	Nice to have for reviews; alternatively, save strategies in a document, rerun them, then either limit by date range or deduplicate.	✓ Has both saving and alerts.
