# Supplementary material for: Preprint pointers from a long COVID scoping review: considerations for source selection and searching
Source: J Can Health Libr Assoc. 2024 Aug 1;45(2):88–97. doi: 10.29173/jchla29741 (PMC11485164; doi:10.29173/jchla29741)
Supplement: Supplementary file 3 [file JCHLA-45-088-s003.pdf]

Appendix 3 - Preprints search strategy in Europe PMC

The Europe PMC search strategy was conducted in 4 parts, run separately, results combined, and then duplicates removed in EndNote. The main database search was peer-reviewed, but this search has not been peer-reviewed.

Please note that search syntax has changed since the development of this search; including the index field TITLE_ABS: which combines both the title and/or abstract fields. (Readers will notice that keywords are repeated below in both the TITLE: OR ABSTRACT: fields, instead.) A single longer search may also work in one search rather than four.

Though Europe PMC initially had the option to use a COVID-19 search string in early 2020, it was no longer available by October 2021. Europe PMC does not have subject headings.

Database: Europe PMC, https://europepmc.org/

Date of search: October 25, 2021. Alerts ran every three months until February 4, 2022.

Limits: reviews or evidence syntheses, English or French language, posted Jan 1, 2019 to Feb 4, 2022.

Europe PMC syntax guide

SRC:PPR	Limit sources to preprints.
"phrase"	Searches for the exact phrase within quotations. (Must be straight quotations).
TITLE:	Title field search.

ABSTRACT:	Abstract field search.
*	Truncation: after a term, any characters can appear to retrieve plurals or varying endings.

Boolean **AND** operators are in bold font to improve readability.

Search 1: Post-COVID-19 condition phrases in the title or abstract, with review terms in the title.

(SRC:PPR) **AND** (TITLE:"long COVID" or TITLE:"long COVID19" or TITLE:"long coronavirus" or TITLE:"post-COVID condition" or TITLE:"post-COVID-19 condition" or TITLE:"chronic COVID condition" or TITLE:"chronic COVID-19 condition" or TITLE:"chronic COVID syndrome" or TITLE:"chronic COVID-19 syndrome" or ABSTRACT:"long COVID" or ABSTRACT:"long COVID19" or ABSTRACT:"long coronavirus" or ABSTRACT:"post-COVID condition" or ABSTRACT:"post-COVID-19 condition" or ABSTRACT:"chronic COVID condition" or ABSTRACT:"chronic COVID-19 condition" or ABSTRACT:"chronic COVID syndrome" or ABSTRACT:"chronic COVID-19 syndrome") **AND** (TITLE:systematic or TITLE:methodologic or TITLE:quantitative or TITLE:integrative or TITLE:collaborative or TITLE:umbrella or TITLE:scoping or TITLE:mapping OR TITLE:synthesis or TITLE:syntheses or TITLE:pooled or TITLE:"meta analysis" or TITLE:"meta analyses" or TITLE:metaanalysis or TITLE:metaanalyses or TITLE:metanalysis or TITLE:metanalyses or TITLE:"technology assessment" or TITLE:HTA or TITLE:HTAs or TITLE:"technology overview" or TITLE:"technology appraisal" or TITLE:"evidence report" or TITLE:"comparative efficacy" or TITLE:"comparative effectiveness" or TITLE:"outcomes research" or TITLE:"relative effectiveness" or

TITLE:"indirect comparison" or TITLE:"indirect comparisons" or TITLE:"treatment comparison" or TITLE:"bayesian comparison" or TITLE:"rapid review")

Search 2: Post-acute sequelae AND COVID terms in the title or abstract, with review terms in the title.

(SRC:PPR) **AND** (TITLE:PASC OR TITLE:"post-acute sequelae" or TITLE:"postacute sequelae" or TITLE:"chronic sequelae" or TITLE:"late sequelae" OR TITLE:"long haul" OR TITLE:"long hauler" or TITLE:"long haulers" OR TITLE:longhaul* OR TITLE:longhauler* OR ABSTRACT:"post-acute sequelae" or ABSTRACT:"postacute sequelae" or ABSTRACT:"chronic sequelae" or ABSTRACT:"late sequelae" OR ABSTRACT:"long haul" OR ABSTRACT:"long hauler" or ABSTRACT:"long haulers" OR ABSTRACT:longhaul* OR ABSTRACT:longhauler*) **AND** (TITLE:"2019-nCoV" OR TITLE:"2019nCoV" OR TITLE:"COVID-19" OR TITLE:"SARS-CoV-2" OR TITLE:"COVID19" OR TITLE:"COVID" OR TITLE:"SARS-nCoV" OR TITLE:"Wuhan coronavirus" OR TITLE:"Coronavirus" OR TITLE:"Corona virus" OR TITLE:"corona-virus" OR TITLE:"corona viruses" OR TITLE:"coronaviruses" OR TITLE:"SARS-CoV" or ABSTRACT:"2019-nCoV" OR ABSTRACT:"2019nCoV" OR ABSTRACT:"COVID-19" OR ABSTRACT:"SARS-CoV-2" OR ABSTRACT:"COVID19" OR ABSTRACT:"COVID" OR ABSTRACT:"SARS-nCoV" OR ABSTRACT:"Wuhan coronavirus" OR ABSTRACT:"Coronavirus" OR ABSTRACT:"Corona virus" OR ABSTRACT:"corona-virus" OR ABSTRACT:"corona viruses" OR ABSTRACT:"coronaviruses" OR ABSTRACT:"SARS-CoV") **AND** (TITLE:systematic or TITLE:methodologic or TITLE:quantitative or TITLE:integrative or TITLE:collaborative or TITLE:umbrella or TITLE:scoping or TITLE:mapping OR TITLE:synthesis or TITLE:syntheses or TITLE:pooled or TITLE:"meta analysis" or TITLE:"meta analyses" or TITLE:metaanalysis or

TITLE:metaanalyses or TITLE:metanalysis or TITLE:metanalyses or TITLE:"technology assessment" or TITLE:HTA or TITLE:HTAs or TITLE:"technology overview" or TITLE:"technology appraisal" or TITLE:"evidence report" or TITLE:"comparative efficacy" or TITLE:"comparative effectiveness" or TITLE:"outcomes research" or TITLE:"relative effectiveness" or TITLE:"indirect comparison" or TITLE:"indirect comparisons" or TITLE:"treatment comparison" or TITLE:"bayesian comparison" or TITLE:"rapid review")

Search 3: Additional post-COVID-19 terms in the title, with review terms in the title.

(SRC:PPR) **AND** (TITLE:"post-COVID" or TITLE:"post-COVID-19" or TITLE:"postCOVID" or TITLE:"postCOVID-19" or TITLE:"post coronavirus") AND (TITLE:sequela* or TITLE:syndrome* or TITLE:disorder* or TITLE:illness* or TITLE:condition* or TITLE:symptom* or TITLE:prognos* or TITLE:followup or TITLE:"follow up" or TITLE:cohort or TITLE:care or TITLE:aftercare or TITLE:recover* or TITLE:rehabilit* or TITLE:clinic* or TITLE:center* or TITLE:centre* or TITLE:survivor*) **AND** (TITLE:systematic or TITLE:methodologic or TITLE:quantitative or TITLE:integrative or TITLE:collaborative or TITLE:umbrella or TITLE:scoping or TITLE:mapping OR TITLE:synthesis or TITLE:syntheses or TITLE:pooled or TITLE:"meta analysis" or TITLE:"meta analyses" or TITLE:metaanalysis or TITLE:metaanalyses or TITLE:metanalysis or TITLE:metanalyses or TITLE:"technology assessment" or TITLE:HTA or TITLE:HTAs or TITLE:"technology overview" or TITLE:"technology appraisal" or TITLE:"evidence report" or TITLE:"comparative efficacy" or TITLE:"comparative effectiveness" or TITLE:"outcomes research" or TITLE:"relative effectiveness" or TITLE:"indirect comparison" or TITLE:"indirect comparisons" or TITLE:"treatment comparison" or TITLE:"bayesian comparison" or TITLE:"rapid review")

Search 4: Additional post-viral terms requiring COVID in the title, with review terms in the title.

(SRC:PPR) **AND** (TITLE:"post-viral" or TITLE:postviral or TITLE:"post virus" or TITLE:postvirus or TITLE:"post-acute" OR TITLE:postacute or TITLE:longterm or TITLE:"long term" or TITLE:"long duration" or TITLE:chronic or TITLE:persist* or TITLE:residual or TITLE:prolonged) **AND** (TITLE:"2019-nCoV" OR TITLE:"2019nCoV" OR TITLE:"COVID-19" OR TITLE:"SARS-CoV-2" OR TITLE:"COVID19" OR TITLE:"COVID" OR TITLE:"SARS-nCoV" OR TITLE:"Wuhan coronavirus" OR TITLE:"Coronavirus" OR TITLE:"Corona virus" OR TITLE:"corona-virus" OR TITLE:"corona viruses" OR TITLE:"coronaviruses" OR TITLE:"SARS-CoV") **AND** (TITLE:systematic or TITLE:methodologic or TITLE:quantitative or TITLE:integrative or TITLE:collaborative or TITLE:umbrella or TITLE:scoping or TITLE:mapping OR TITLE:synthesis or TITLE:syntheses or TITLE:pooled TITLE:"meta analysis" or TITLE:"meta analyses" or TITLE:metaanalysis or TITLE:metaanalyses or TITLE:metanalysis or TITLE:metanalyses or TITLE:"technology assessment" or TITLE:HTA or TITLE:HTAs or TITLE:"technology overview" or TITLE:"technology appraisal" or TITLE:"evidence report" or TITLE:"comparative efficacy" or TITLE:"comparative effectiveness" or TITLE:"outcomes research" or TITLE:"relative effectiveness" or TITLE:"indirect comparison" or TITLE:"indirect comparisons" or TITLE:"treatment comparison" or TITLE:"bayesian comparison" or TITLE:"rapid review") **NOT** ("long-term care" OR "longterm care")
