# Supplementary material for: Preprint pointers from a long COVID scoping review: considerations for source selection and searching
Source: J Can Health Libr Assoc. 2024 Aug 1;45(2):88–97. doi: 10.29173/jchla29741 (PMC11485164; doi:10.29173/jchla29741)
Supplement: Supplementary file 4 [file JCHLA-45-088-s004.pdf]

Appendix 4 - Preprint considerations throughout the review

The following are considerations for preprints during a scoping or other review, gleaned from the lessons learned from our long COVID scoping review.

Protocol development

1. **Inclusion criteria:** The protocol should have clear inclusion criteria and methods for if and how preprints will be included. If preprints are excluded, the methods section should provide justification.
2. **Strategy development and size estimates:** Conduct test searches early on to estimate preprint results, plan the impact on timelines, and discuss options for the search approach.
    a. To echo Cochrane,[49] review teams should seek search guidance from an information specialist or medical librarian with experience supporting reviews and preprint searching.
3. **Labelling:** Clearly identify preprints throughout the project with labels in the citation,[11] but not so that duplicate detection is affected. Readers may be unfamiliar with some preprint server names.
4. **Publication policies:** A few journals have policies against citing preprints, or have restrictions for papers that have been published as a preprint;[50] check this up front.

Searching preprints

1. **Source selection:** Determine what preprint servers will be covered by your main database search. Consider adding a separate preprint aggregator that allows for advanced searching and batch export, such as Europe PMC.

    a. For rapid COVID-19 searches on specific diseases or genes, preVIEW (reviewed separately[16]) is another free preprint aggregator.

2. **Plan translation time early:** If adding a separate aggregator, start translation early and plan for additional time.
    a. Parallel the main database strategy where possible. If you have study design or other limits for the main search, apply the same limits.
    b. Polyglot may have automatic search translation for a similar syntax, then use Find & Replace in a word processor for remaining changes.
    c. If an exact translation of the search is not possible, a broader search is more comprehensive for a systematic review. Some review types may have the flexibility to narrow the preprint search.
    d. Some preprint sources (such as Europe PMC) will search the full text of preprints by default. Consider limiting keyword searches to the title and abstract fields.
    e. In case of a large number of results, early planning helps decide if there's time to screen the full set. There's no standard approach for narrowing preprint searches. Options to discuss with the team include searching only title and abstract fields, searching fewer servers, limiting to preprints without a publication yet (an option in Europe PMC), limiting by date, or limiting by study designs.

Managing, evaluating, and tracking preprints

1. **Publication check:** Add a publication check for all included preprints near the end of the report. This will confirm that the review is based on the latest version of the studies and that none are double counted.
    a. Create a separate tracking list of preprint & publication pairs if it's helpful.

    b. If the preprint server doesn't link to a final publication, also conduct an Internet search of the title and author names.

2. **Sensitivity analysis:** If preprints are included in a systematic review, Clyne et al. (2021) suggest a sensitivity analysis of the impact of including preprints on the conclusion.[10]
